# The specific mitochondrial unfolded protein response in fast- and slow-twitch muscles of high-fat diet-induced insulin-resistant rats

**DOI:** 10.3389/fendo.2023.1127524

**Published:** 2023-03-16

**Authors:** Can Li, Nan Li, Ziyi Zhang, Yu Song, Jialin Li, Zhe Wang, Hai Bo, Yong Zhang

**Affiliations:** ^1^ Tianjin Key Laboratory of Exercise Physiology and Sports Medicine, School of Exercise and Health, Tianjin University of Sport, Tianjin, China; ^2^ Department of Military Training Medicines, Logistics University of Chinese People’s Armed Police Force, Tianjin, China

**Keywords:** insulin resistance, skeletal muscle fiber type, UPRmt, MOTS-c, epigenetic modification

## Abstract

**Introduction:**

Skeletal muscle insulin resistance (IR) plays an important role in the pathogenesis of type 2 diabetes mellitus. Skeletal muscle is a heterogeneous tissue composed of different muscle fiber types that contribute distinctly to IR development. Glucose transport shows more protection in slow-twitch muscles than in fast-twitch muscles during IR development, while the mechanisms involved remain unclear. Therefore, we investigated the role of the mitochondrial unfolded protein response (UPRmt) in the distinct resistance of two types of muscle in IR.

**Methods:**

Male Wistar rats were divided into high-fat diet (HFD) feeding and control groups. We measured glucose transport, mitochondrial respiration, UPRmt and histone methylation modification of UPRmt-related proteins to examine the UPRmt in the slow fiber-enriched soleus (Sol) and fast fiber-enriched tibialis anterior (TA) under HFD conditions.

**Results:**

Our results indicate that 18 weeks of HFD can cause systemic IR, while the disturbance of Glut4-dependent glucose transport only occurred in fast-twitch muscle. The expression levels of UPRmt markers, including ATF5, HSP60 and ClpP, and the UPRmt-related mitokine MOTS-c were significantly higher in slow-twitch muscle than in fast-twitch muscle under HFD conditions. Mitochondrial respiratory function is maintained only in slow-twitch muscle. Additionally, in the Sol, histone methylation at the ATF5 promoter region was significantly higher than that in the TA after HFD feeding.

**Conclusion:**

The expression of proteins involved in glucose transport in slow-twitch muscle remains almost unaltered after HFD intervention, whereas a significant decline of these proteins was observed in fast-twitch muscle. Specific activation of the UPRmt in slow-twitch muscle, accompanied by higher mitochondrial respiratory function and MOTS-c expression, may contribute to the higher resistance to HFD in slow-twitch muscle. Notably, the different histone modifications of UPRmt regulators may underlie the specific activation of the UPRmt in different muscle types. However, future work applying genetic or pharmacological approaches should further uncover the relationship between the UPRmt and insulin resistance.

## 1 Introduction

As the largest metabolic tissue in the body, skeletal muscle accounts for approximately 40% of body weight and has the ability to utilize large amounts of blood sugar in the body (1). At rest, skeletal muscle consumes over 20% of the body’s energy expenditure (2), while during exercise, skeletal muscle is responsible for at least 95% of the additional energy consumption (3). In addition, the consumption rate of postprandial blood glucose in skeletal muscle is more than 80%~90% (4). Therefore, skeletal muscle is important for systemic metabolic function and has the potential to serve as a therapeutic target for metabolic diseases.

As a heterogeneous tissue, mammalian skeletal muscle is composed of different types of muscle fibers, and different muscle fibers have specific metabolic characteristics (5). Generally, slow-twitch muscle composed of type I and IIa fibers has a relatively slow contraction rate, high glucose/lipid oxidation ratio and high densities of mitochondria and capillaries. Fast-twitch muscle consists of type IIx and IIb fibers, which are characterized by a fast contraction speed, relatively high glycolysis rate and few mitochondria (5). These disparities may influence the response of muscles to certain physiological and pathological challenges, such as exercise (6), aging (7) and insulin resistance (8). Mitochondria from slow-twitch muscles have an approximately twofold higher H_2_O_2_ scavenging capacity than mitochondria from fast-twitch muscles (9). Slow-twitch muscles also show higher activities of antioxidant enzymes than fast-twitch muscles (10). Thus, slow-twitch muscles usually receive more protection than fast-twitch muscles in oxidative stress-related diseases (11). Stuart et al. (12) showed positive correlations between the proportion of type I fibers in muscle and whole-body insulin sensitivity. In obese and type 2 diabetes (T2D) individuals, the glucose-handling capacity of slow oxidative muscle fibers was significantly higher than that of fast glycolytic muscle fibers (8). In summary, glucose transport shows more protection in slow-twitch muscles than in fast-twitch muscles during insulin resistance (IR) development, while the mechanisms involved remain unclear.

Mitochondria are one of the most important organelles in mammalian skeletal muscle cells, and mitochondrial homeostasis is essential for the normal function of skeletal muscle. Mitochondrial dysfunction may lead to metabolic disorders, loss of muscle mass and contractile dysfunction. Therefore, mitochondria have developed a series of mechanisms to maintain their homeostasis, including antioxidant machinery, fission and fusion, mitochondrial biogenesis, mitophagy (13) and the mitochondrial unfolded protein response (UPRmt) (14). Evidence suggests that insulin sensitivity in skeletal muscle is closely related to mitochondrial homeostasis. A previous study showed that the inhibition of mitochondrial oxidative stress partly preserved insulin sensitivity in human and rodent muscles (15). Aberrant mitochondrial fission is associated with mitochondrial dysfunction and insulin resistance in skeletal muscle (16). Additionally, mitochondrial respiration is decreased in the skeletal muscle of individuals with T2D (17). In isolated insulin-resistant rat skeletal muscle cells, the restoration of insulin-stimulated glucose uptake induced by melatonin is accompanied by preserved mitochondrial respiration (18). Overall, approaches to enhance mitochondrial homeostasis may improve insulin sensitivity in skeletal muscle.

The UPRmt is an adaptive transcriptional response that was initially described as a mechanism for cells to maintain mitochondrial protein homeostasis during mitochondrial dysfunction, as these organelles are constantly importing and processing proteins in an unfolded state. Hoogenraad et al. (19) described the pathway in rat hepatoma cells in which mitochondrial proteostasis was disturbed by ethidium bromide (EB) treatment, characterized by the accumulation of unfolded proteins in mitochondria. Zhao et al. (20) found similar results in mitochondria that were mildly stressed by overexpression of ΔOTC, a mutated ornithine transcarbamylase that cannot properly fold within mitochondria. Both of their studies confirmed the restoration of mitochondrial homeostasis and cellular status after UPRmt activation. The UPRmt is a mitochondrial stress response by which mitochondria initiate the transcriptional activation programs of mitochondrial chaperone proteins and proteases encoded by nuclear DNA to maintain proteostasis (21). As a stress-triggered process, UPRmt activation ultimately leads to mitochondrial functional recovery (22), metabolic adaptations (22), and innate immunity (23) at the cellular level.

Previous studies have shown that activation of the UPRmt in multiple tissues exhibits protective roles in a variety of diseases. Wang et al. (24) reported that UPRmt induced by doxycycline has protective effects on cardiac infarction, but this protection is absent in Atf5^-/-^ mice. This research confirmed the protective effect of ATF5-dependent UPRmt in myocardial disease. Another study obtained similar results, confirming that the UPRmt could be activated in the hearts of mice subjected to chronic hemodynamic overload, and further boosting UPRmt with nicotinamide riboside in cardiomyocytes *in vitro* or in hearts *in vivo* significantly ameliorated the decline in mitochondrial respiration induced by these stresses (25). In addition, Gariani et al. (26) demonstrated that in long-term high-fat high-sucrose-fed animals, nicotinamide riboside could increase sirtuin-mediated UPRmt activation, triggering an adaptive process to increase hepatic β-oxidation and mitochondrial complex content and activity. A recent study demonstrated that UPRmt markers were elevated in acute insulin resistance in skeletal muscle induced by a high-fat diet (27). This research indicated that mitochondrial damage from a high-fat diet (HFD) induced the UPRmt as a stress response in skeletal muscle.

As mentioned above, different types of muscle fibers contribute distinctly to the development of insulin resistance. It is unclear whether the UPRmt is involved in this muscle type-dependent metabolic adaptation. Therefore, the purpose of this study was to investigate the role of UPRmt in the distinct resistance of two types of muscle in IR. The soleus (Sol) and tibialis anterior (TA) were selected to represent slow- and fast-twitch muscles, respectively.

## 2 Materials and methods

### 2.1 Animals and sample collection

Twenty-four male Wistar rats (8 weeks old) were purchased from Beijing Vital River Laboratory Animal Technology Co. Ltd. and then migrated into air-conditioned polypropylene cages under a 12-h light/dark cycle in our SPF-grade facility. The room temperature was maintained at 24~27°C and 40-45% relative humidity. After 1 week of acclimatization, rats were randomly divided into two groups: (1) the control group (n=12) was fed a normal chow diet (14 kcal% from fat, mainly consisting of corn oil, 66 kcal% from carbohydrate and 20 kcal% from protein), and (2) the HFD group (n=12) was fed a high-fat diet (60 kcal% from fat, mainly consisting of lard, 20 kcal% from carbohydrate and 20 kcal% from protein) for 18 weeks. The chow diet (HFK, #1032) and the high-fat diet (HFK, #H10060) were purchased from Beijing HFK Bioscience Co., Ltd. All rats had free access to food and drinking water. At the end of the final week, 8 rats from each group were selected for FBG and OGTT tests after 6 h of fasting. Six hours after those tests, these rats were euthanized under anesthesia induced by sevoflurane followed by intraperitoneal injection of pentobarbital sodium. Skeletal muscle tissues and blood samples were harvested immediately for subsequent experiments. Simultaneously, 4 rats from each group were selected for use only in the hyperinsulinemic–euglycemic clamp test and were then killed by euthanasia. Due to their predominant muscle fiber type in whole muscle, the Sol muscle is representative of slow oxidative muscle, and the TA muscle is representative of fast glycolytic muscle. All animals received proper care, and all animal experiments were approved and supervised by the Animal Ethics Committee of the Tianjin University of Sport in accordance with the National Research Council’s Guide for the Care and Use of Laboratory Animals.

### 2.2 Fasting insulin test, fasting blood glucose test and oral glucose tolerance test

The FIN test was used to measure the fasting blood insulin concentration of the rats. This test was performed by using the blood sample collected from the last step. The fasting insulin levels in the plasma were determined using an assay kit (CUSABIO, FIN Elisa kit) according to the manufacturer’s instructions. Before the FBG and OGTT, the rats were fasted for 6 h. An FBG test was used to assess the fasting blood glucose level. Blood samples were collected by pricking the rat tail tip with a needle. Blood glucose test strips (Roche Accu-Check Active) were used to determine the FBG of each rat. Homeostasis model assessment of insulin resistance (HOMA-IR) was calculated based on the equation HOMA-IR=serum insulin (mmol/L)×blood glucose (mmol/L)/22.5 (28). The OGTT was used to evaluate the glucose tolerance of the rats. A 50% glucose Solution was infused by intragastric administration at a dose of 2 g of glucose per 1 kg of body weight, and blood glucose was measured every half hour after intragastric administration. This procedure lasted for 2.5 h. GraphPad Prism 8 was used to establish the OGTT curve. Blood glucose values at each time point were recorded, and continuous curves were drawn. The area under the curve (AUC) was calculated to evaluate the insulin sensitivity of rats.

### 2.3 Hyperinsulinemic-euglycemic clamp test

Four rats were selected from each group for the hyperinsulinemic–euglycemic clamp test. Anesthesia for selected rats was induced by sevoflurane, followed by intraperitoneal injection of pentobarbital sodium after 6 h of fasting. The right jugular and left carotid were exposed for the following cannulation: a t-branch for glucose and insulin infusion was catheterized into the right jugular, and an indwelling needle was catheterized into the left carotid for blood sample collection. Recombinant human insulin (Humulin R) was infused by a peristaltic pump at a fixed flow rate of 5 mU/kg/min; glucose was infused by another peristaltic pump, and the infusion rate (GIR) was adjusted promptly to maintain blood glucose at ~5.0 mM. Carotid blood was collected for the blood glucose test every 5 min for 2 h. Insulin sensitivity was evaluated by the GIR during the last 30 min of the experiment and quantified by the AUC of the data collected from this duration. To avoid the unknown influences exerted by a constant infusion of exogenesis insulin lasting for 2 h, these rats were then killed by euthanasia after the clamp test and no longer used for other experiments.

### 2.4 Mitochondrial isolation from skeletal muscles

The mitochondrial isolation procedure was based on the sucrose step density gradient centrifugation method (29). The isolation procedure is briefly described as follows: 300 mg of skeletal muscle tissue was taken from each muscle collected in the last step for mitochondrial isolation. The muscle tissue was first cut into very small pieces and then transferred into a Dounce homogenizer for gentle homogenization. This whole step was performed in ice-cold MS homogenization buffer (210 mM mannitol, 70 mM sucrose, 5 mM Tris-HCl, 1 mM EDTA, pH 7.5) as quickly as possible. The suspension was centrifuged at 1000 × g and 4°C for 10 min, and then the supernatant was transferred into another tube for a 2nd centrifugation at 12,000 × g and 4°C for 10 min. The precipitate was crude skeletal muscle mitochondria. The final pellet was resuspended in a volume and buffer appropriate for subsequent work. The mitochondrial suspension was carefully applied on top of the sucrose step density gradient Solution (15 mL of 1.0 M sucrose gradient Solution over 15 mL of 1.5 M sucrose gradient Solution, 10 mM Tris-HCl, 1 mM EDTA, pH 7.5) in Ultra-Clear centrifuge tubes. The tubes were centrifuged at 50,000 × g for 20 min. The purified mitochondria formed a layer at 1.0 M/1.5 M sucrose interface. The final dilution and resuspension of the purified mitochondria from the last step were performed according to previous research (29). Finally, purified and intact mitochondria were prepared for other experiments.

### 2.5 Mitochondrial respiration

Skeletal muscle mitochondrial respiratory function was assessed by an Oroboros Oxygraph-2k (Oroboros Instruments, 10023-01) as previously described (30). In brief, the MiR05-Kit (Oroboros Instruments, 60101-01) served as the mitochondrial respiration medium. Two milliliters of medium was initially injected into the system, and the O_2_ calibration procedure for adequate O_2_ dissolution was immediately performed. Then, 100 μg of mitochondria was added to the reaction system after O_2_ calibration, and a mixture of malate (final conc. 2 mM) and glutamate (final conc. 10 mM) was added to the system when the O_2_ consumption curve became steady. This phase of O_2_ consumption is state 2 respiration. When the curve plateaued, ADP (final conc. 0.1 mM) was added to the system; the O_2_ consumption in this phase represents state 3 respiration (ST3). The final steady curve of O_2_ consumption followed by ADP exhaustion represents state 4 respiration (ST4). The respiratory control rate (RCR) was calculated as ST3/ST4.

### 2.6 Western blotting and real-time qPCR

Western blotting (WB) was used to determine the expression of recognized glucose transport markers, UPRmt markers and MOTS-c in this study. The experiments were performed under the standard WB procedure, briefly as below. A 100-mg muscle sample cut into very small pieces or ~5 mg of isolated mitochondria was transferred into a Dounce homogenizer (200-μl EP tube for mitochondria) with 1 ml (0.05 ml for mitochondria) of RIPA lysis buffer (Beyotime, P0013B) containing 10 μl (1 μl for mitochondria) of protease and phosphatase inhibitor cocktail (Thermo Scientific™, #78445) for thorough homogenization (ultrasonication for mitochondria). The supernatant was then collected after centrifugation for protein concentration determination by using a BCA protein assay kit (Beyotime, P0012). Denaturation was performed with protein loading buffer (Solarbio, P1040). Denatured proteins were separated by SDS−PAGE electrophoresis and transferred to 0.22-μm PVDF membranes (Millipore, Billerica). Immobilon Western Chemiluminescent HRP Substrate (Millipore, WBKLS0500) was used for target protein detection after blocking and primary/secondary antibody incubations of PVDF membranes. Adobe Photoshop CC was used to quantify the luminance intensity of the protein bands. The following primary antibodies were used: Insulin Receptor beta (Abcam, ab69508); IRS-1 (CST, #2382); Phospho-IRS-1 Ser307 (CST,#2381); AS160 (CST, 2670); Phospho-AS160 T642 (CST, 8881); Glut4 (Abcam, ab33780); CHOP (CST, 2895); ATF5 (Abcam, ab184923); HSP60 (CST, 4870S); ClpP (Abcam, ab124822); COX4 (CST, 4850S);β-tubulin (CST, 2146S) and Mots-c (FabGennix, MOTSC-101AP).

Real-time qPCR was chosen to assess the relative fold change in *MOTS-c*. Total RNA was extracted from ~50 mg of tissue by using the Dynabeads mRNA Purification Kit (Invitrogen™, #61006), and the operation was performed according to the manufacturer’s instructions. RNA was reverse transcribed using a High-Capacity RNA-to-cDNA™ kit (Thermo Scientific™, #4368813), and quantitative real-time PCR was performed on a 96-well PCR device (7500 Real-Time PCR System, Applied Biosystems™) by using SYBR Green Select Master Mix (Applied Biosystems™, #4472919). All procedures were performed following the manufacturers’ instructions. *β-Actin* was used to normalize the expression levels of target genes, and the relative expression of *MOTS-c* was analyzed using the 2^−ΔΔCt^ method. The specific sequence information for the primers used in this study is as follows: *MOTS-c* Forward, 5′-GACACCTTGCCTAGCCACAC-3′; *MOTS-c* Reverse, 5′-TGGCTGGCACGAAATTTACCA-3′; *β-Actin* Forward, 5′-CCGTAAAGACCTCTATGCCAACA-3′; *β-Actin* Reverse, 5′-TATCCATTCTCAAGAGCAGCGAAAG-3′.

### 2.7 Chromatin immunoprecipitation

In this study, the two kinds of histone modifications on the ATF5 promoter were detected by ChIP. ChIP experiments and the subsequent purification of cross-linked DNA were performed using a SimpleChIP^®^ Enzymatic Chromatin IP Kit (CST, 9003), and all procedures were performed according to the manufacturer’s instructions. Anti-H3K4me3 (CST, 9751) and anti-H3K27me3 (CST, 9733) were used to measure the corresponding histone methylation at the *ATF5* promoter region, and rabbit mAb IgG (CST, 3900) was used as the negative control. The purified DNA samples from immunoprecipitation were further quantified by RT-qPCR (7500 Real-Time PCR System, Applied Biosystems™). Values were normalized to each individual input control. The primers were specifically designed for the *ATF5* gene promoter region, and the sequences were as follows: *ATF5* promoter Forward, 5′- AGGAGGACCATAGGCATTG-3′; *ATF5* promoter Reverse, 5′- GCTAGACAGGCATTCTACCAC -3′.

### 2.8 Statistical analysis

The values in this study are presented as the mean ± SD. The data were analyzed using independent T tests, two-way ANOVA and two-way ANOVA with repeated measures, followed by a *post hoc* Bonferroni’s multiple comparison tests, as appropriate. Analyses of the main effects (muscle type) and interaction effects (muscle type×diet protocol) were also conducted. Descriptive and analytical statistics were examined with SPSS v26.0 software. Results with *p* values < 0.05 were considered statistically significant.

## 3 Results

### 3.1 A high-fat diet causes systemic insulin resistance

**Figure 1:**
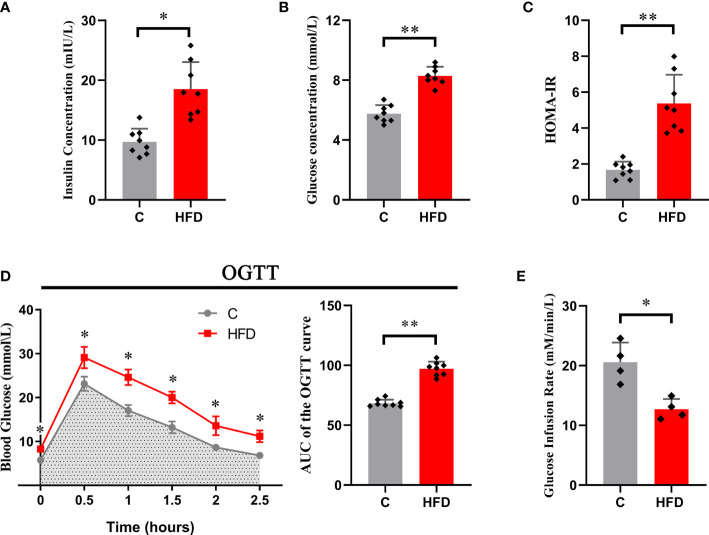
Whole-body insulin resistance induced by HFD. Each graph represents **(A)** fasting blood insulin concentration; **(B)** fasting blood glucose; **(C)** HOMA-IR; **(D)** the original OGTT curve and its AUC; and **(E)** glucose infusion rate. (*p<0.05; *p<0.01; n=8 in each group in graphs **(A–D)**, n=4 in each group in graph **E**).

After 18 weeks of HFD feeding, rats in the HFD group showed a significant increase in blood insulin concentration (6.98 ± 2.08 *vs.* 15.54 ± 4.59, *p*<0.01) and fasting blood glucose (5.75 ± 0.58 *vs.* 8.29 ± 0.6, *p*<0.05) compared with those in the control group (**Figures 1A, B**). Consistent with this, the HOMA-IR index was significantly increased in HFD rats compared with the controls (1.67 ± 0.46 *vs.* 5.37 ± 1.59, *p*<0.01) (**Figure 1C**). In the hyperinsulinemic-euglycemic clamp test, HFD rats showed a significantly decreased GIR (20.56 ± 3.31 *vs.* 12.71 ± 1.71, *p*<0.01) (**Figure 1E**). The AUC of the OGTT curve reflected that the glucose tolerance of HFD rats was much lower than that of the control group (97.03 ± 2.89 *vs.* 68.44 ± 2.06, *p*<0.01) (**Figure 1D**). These results indicate that long-term (18 weeks) HFD consumption could induce whole-body insulin resistance in rats.

### 3.2 A high-fat diet causes impairment of glucose transport specifically in fast-twitch muscle

**Figure 2:**
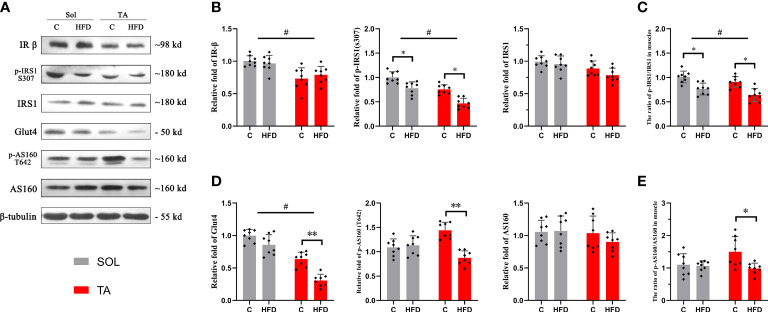
Insulin and glucose transport in fast- and slow-twitch muscles after HFD intervention. **(A)** The original western blotting results of phosphorylated IRS1 (S307), IRS1, IR-β, phosphorylated AS160 (T642), AS160 and Glut4 in muscle lysates. **(B)** Quantification of relative expression of p-IRS1 (S307), IRS1 and IR-β protein in each group. **(C)** The ratio of the relative expression of p-IRS1 and total IRS1. **(D)** Quantification of relative expression of the p-AS160 (T642), AS160 and Glut4 protein in each group. **(E)** The ratio of the relative expression of p-IRS1/IRS1. (*p<0.05; **p<0.01; and #p<0.05 when comparison was conducted between muscles; n=8 for each group).

The Sol and TA were chosen for investigating the process of HFD-induced adaptation/deterioration of different muscles. To evaluate the impairment of insulin signaling and glucose transport in slow- and fast-twitch muscles in response to HFD, we tested the protein levels of insulin receptor-β (IR-β), p-IRS1, IRS1, Glut4, AS160 and p-AS160, and the results were as follows: first, the results of the two-way ANOVA of IR-β expression showed a significant main effect of muscle type (*p*<0.05) but no main effect of dietary intervention. The IR-β content was significantly higher in Sol than that in TA muscle (**Figures 2A, B**). However, the expression of p-IRS1, the active form of IRS1, and the p-IRS1/IRS ratio showed a significant decrease (*p*<0.05) in both Sol and TA in response to the HFD (**Figures 2A–C**). Moreover, there was no significant interaction effect between muscle type and dietary intervention. Second, we found that the expression of Glut4 in TA muscle was markedly lower in the HFD group than in the C group (*p*<0.01) (**Figures 2A, D**). Similarly, in comparison to the C group, the expression of p-AS160 (T642) in the HFD group was significantly downregulated 0.40-fold (*p*<0.05) in the TA (**Figures 2A, D**). Consistent with this, the ratio of p-AS160/AS160 in the TA also decreased 0.36-fold (*p*<0.05) in the control group compared to the HFD group (**Figures 2A, E**). However, the expression of Glut4, AS160 and p-AS160 showed no significant changes in the Sol (**Figure 2**). The results of the two-way ANOVA of Glut4 expression showed a significant main effect of muscle type (*p*<0.05) and a main effect of dietary intervention (*p*<0.05). Moreover, there was a significant interaction effect between muscle type and dietary intervention (*p*<0.05). These results revealed that the impairments in glucose transport induced by a HFD occur specifically in fast-twitch muscles.

### 3.3 A high-fat diet causes mitochondrial respiratory dysfunction in fast-twitch muscle

**Figure 3:**
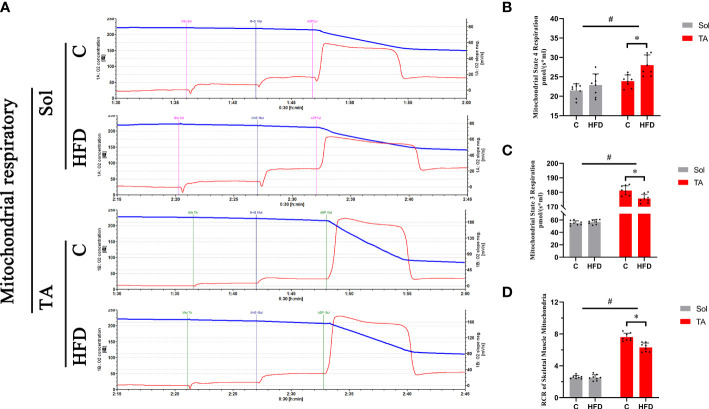
Mitochondrial respiratory functions in fast- and slow-twitch muscles after HFD intervention. Traces of mitochondrial O_2_ consumption **(A)**, Mitochondrial State 4 **(B)** and State 3 **(C)** respiration and the respiratory control rate **(D)** in the Sol and TA under stimulation with the respiratory chain substrate (malate+glutamate) are shown. (*p<0.05; n=8 for each group).

To investigate the alterations in mitochondrial respiratory function in different muscles under HFD conditions, mitochondrial respiration was measured by an Oroboros Oxygraph-2k. The results showed no significant changes in mitochondrial respiration in Sol muscle after HFD feeding. In TA mitochondria, a significant decrease in ST3 and an increase in ST4 were observed in HFD rats compared with control rats (*p*<0.05), and the RCR (ST3/ST4) was significantly decreased in this comparison (*p*<0.05) (**Figure 3**). Two-way ANOVA revealed a significant main effect of muscle type (*p*<0.05) for ST3 and RCR; these two parameters were significantly higher in the TA than in Sol muscle. In summary, mitochondrial respiratory function was decreased only in the TA but not in the Sol muscle after long-term HFD feeding.

### 3.4 ATF5-dependent UPRmt is preferentially activated by HFD in slow-twitch muscle

**Figure 4:**
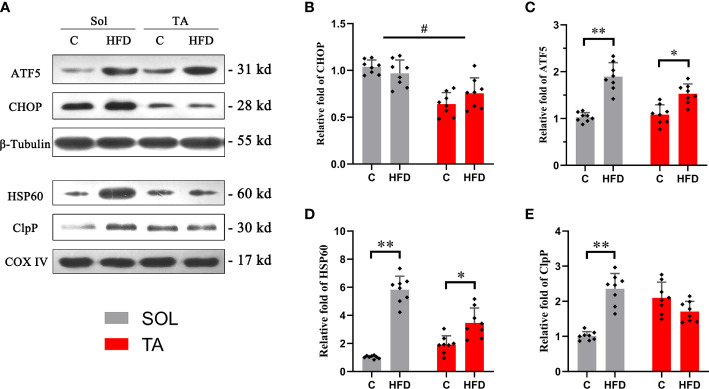
The relative expression of UPRmt markers in Sol and TA muscles. **(A)** The original western blots of CHOP and ATF5 from muscle lysates; HSP60 and ClpP from isolated mitochondria. **(B, C)** Quantification of the relative expression of CHOP and ATF5 in Sol and TA in each group. **(D, E)** Quantification of the relative expression of HSP60 and ClpP in Sol and TA in each group. (*p<0.05; **p<0.01; and #p<0.05 when comparison was conducted between muscles; n=8 for each group).

Western blotting was used to measure the expression of the identified UPRmt markers. ATF5 and CHOP are located in multiple regions in the cell; thus, the expression levels of these proteins were measured in muscle homogenates. HSP60 and ClpP are located in the mitochondrial matrix, so these proteins should be measured in isolated mitochondria. The results showed that the expression level of ATF5 was upregulated by 0.84-fold (*p*<0.05) in HFD rats compared to the controls in Sol muscle and was upregulated by 0.43-fold (*p*<0.05) in TA muscle (**Figures 4A, C**). While two-way ANOVA revealed a significant interaction effect between muscle types and dietary intervention for the expression of ATF5. No significant changes in the expression level of CHOP were observed in either Sol or TA muscle in HFD rats compared to the controls (**Figures 4A, B**). Two-way ANOVA revealed a significant main effect of muscle type (*p*<0.05), the expression of CHOP in Sol was significantly higher than that in TA muscle. Moreover, for the expression of CHOP, no significant interaction effect between muscle types and dietary intervention was observed. Regarding Sol mitochondrial proteins, HFD rats showed markedly upregulated expression of HSP60 by 4.68-fold (*p*<0.01) and of ClpP by 1.33-fold (*p*<0.01) compared with control rats (**Figures 4A, D, E**). In TA mitochondria, only the expression of HSP60 was upregulated 1.44-fold (*p*<0.05) in HFD rats compared to control rats (**Figures 4A, D**). No significant changes in ClpP were observed in TA mitochondria after HFD consumption. Notably, two-way ANOVA revealed no significant main effect of muscle type for the expression of HSP60 and ClpP, but it indicated a significant interaction effect between muscle type and dietary intervention for both HSP60 and ClpP levels (*p*<0.05). These results indicated that the activation of the UPRmt in Sol muscle was more profound than that in TA muscle after HFD intervention.

### 3.5 The expression of MOTS-c decreased after HFD feeding specifically in fast TA muscle

**Figure 5:**
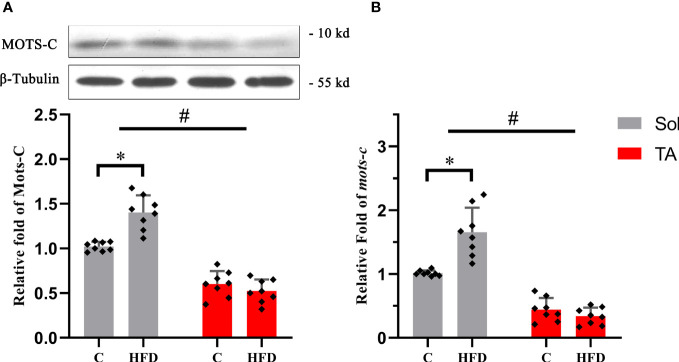
The relative protein and mRNA expression levels of MOTS-c in slow- and fast-twitch muscles among all groups. **(A)** The protein expression level of MOTS-c in the Sol and TA muscles among the groups. **(B)** Relative fold change in MOTS-c mRNA levels in the Sol and TA muscles among the groups. (*p<0.05 and #p<0.05 when comparison was conducted between muscles; n=8 for each group).

MOTS-c is a mitochondria-derived peptide that can participate in regulating glucose metabolism. Our results showed that the expression of MOTS-c increased by 0.37 times (*p*<0.05) in Sol muscle after HFD feeding. Consistent with the protein expression, compared with the control group, the mRNA level of *MOTS-c* in the Sol muscle of HFD-fed rats was significantly increased by 0.62 times (*p*<0.05). In the TA, there was no significant difference in the expression of MOTS-c protein or mRNA after HFD feeding (**Figure 5**). In this section, two-way ANOVA revealed a significant main effect of muscle type (*p*<0.05) for the protein and mRNA levels of MOTS-c. Moreover, it showed a significant interaction effect of muscle type and dietary intervention for both the protein and mRNA levels of MOTS-c (*p*<0.05).

### 3.6 Epigenetic modulation, which is associated with transcription promotion, occurred only in slow-twitch muscle after HFD feeding

**Figure 6:**
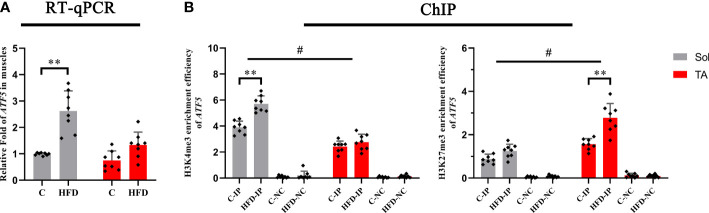
The transcription level and histone methylation at the promoter region of ATF5. **(A)** The transcription level of ATF5 in Sol and TA muscles among the groups; **(B)** The H3K4me3 and H3K27me3 enrichment rate of the ATF5 promoter in Sol and TA muscles among the groups. **p<0.01; and #p<0.05 when comparison was conducted between muscles; n=8 for each group; IP, immunoprecipitation; NC, negative control).

Histone methylations, including H3K4me3 and H3K27me3, have been proven to be involved in regulating the UPRmt (31, 32), in which H3K4me3 has the effect of activating transcription, whereas H3K27me3 inhibits transcription. First, we tested the transcription levels of *ATF5* in the Sol and TA muscles of the HFD group and control group. We found that the mRNA level of *ATF5* in the Sol of HFD rats significantly increased by 1.54 times compared with that of the control group (*p*<0.01) (**Figure 6A**). Furthermore, the ChIP assay results showed that compared with that in the control rats, the enrichment of H3K4me3 in the promoter region of *ATF5* in Sol was significantly upregulated by 0.5-fold in HFD rats (*p*<0.01) (**Figure 6B**). The enrichment of H3K27me3 in the promoter region of *ATF5* in the TA muscle of HFD-fed rats was significantly increased by 0.77-fold (*p*<0.01) (**Figure 6B**). Notably, for these two opposite epigenetic modulations, the findings showed both a significant main effect of muscle type (*p*<0.05) and a significant interaction effect between muscle type and dietary intervention (*p*<0.05). In summary, both the higher enrichment efficiency of H3K4me3 at the ATF5 promoter region in Sol muscle and the higher enrichment efficiency of H3K27me3 at the ATF5 promoter region in TA muscle may contribute to the more profoundly upregulated ATF5 in Sol muscle than in TA muscle.

## 4 Discussion

As a heterogeneous tissue, skeletal muscle differs with respect to its different muscle fiber types in response to physiological and pathological stresses. In the present study, a HFD-induced insulin resistance model was established, and we found that the impairment in glucose transport in fast-twitch muscle was more pronounced than that in slow-twitch muscle. The main novelty of this paper is to reveal that UPRmt is mainly activated in the Sol after HFD feeding, not in the TA. Along with the activation of the UPRmt, the maintenance of mitochondrial respiration function and the upregulated expression of MOTS-c, a mitokine involved in regulating glucose metabolism, were observed only in the Sol. Further ChIP experiments revealed that the higher histone methylation H3K4me3 at the *ATF5* promoter region in Sol and H3K27me3 at the *ATF5* promoter region in TA may contribute to the more pronounced activation of the UPRmt in Sol muscle.

After 18 weeks of HFD feeding, we tested the status of glucose metabolism in all of the rats. Following a previous study that discussed the establishment of an insulin resistance rat model induced by long-term high-fat diet feeding (33), we selected the HOMA-IR (28), OGTT and hyperinsulinemic-euglycemic clamp test (34) to evaluate systemic glucose metabolism in rats. Our results indicated that the HFD rats developed systemic insulin resistance.

Different types of muscle fibers contribute differently to adaptation or deterioration under metabolic stress, such as a HFD (35). Through further investigation of glucose metabolism in slow- and fast-twitch skeletal muscles, our results showed that the AS160-Glut4 axis, the key player in the regulation of glucose transport (36), was significantly decreased only in TA muscle after HFD feeding. These results imply that fast-twitch muscle is more susceptible to HFD than slow-twitch muscle and is more likely to suffer impairment in glucose metabolism. Meanwhile, slow-twitch muscles may be more tolerant to HFD. In line with our results, a previous study indicated that short-chain fatty acyl CoA dehydrogenase activity was elevated after 8 weeks of HFD feeding in Sol muscle but was not changed in TA muscle (37). For HFD versus control diet-fed rats, the glucose uptake in insulin-stimulated single fibers was significantly (*p* < 0.05) lower for type II but not type I fibers (38). A series of studies conducted by Cartee (35, 39) found that 2 weeks of HFD feeding could cause a significant decrease in Glut4 expression in type IIb fibers, as well as insulin-stimulated glucose uptake in multiple fast type II fibers but not type I fibers. In addition, the insulin-stimulated elevation of p-AS160 (S704) was blunted in type IIx and IIbx fibers in insulin-resistant rats (39). Consistently, in this study, the disparities in the expression of proteins involved in glucose transport show that the glucose transport of slow-twitch muscle remains almost unaltered after HFD intervention, but glucose transport of fast-twitch muscle was significantly impaired.

As a stress-triggered mitochondrial protection response, the UPRmt has gained much attention in recent decades. The canonical UPRmt transcriptional axis is activated upon mitochondrial protein misfolding/aggregation in the mitochondrial matrix. In addition, the UPRmt sirtuin axis and the UPRmt estrogen receptor alpha axis are likely highly complementary to the canonical UPRmt transcriptional axis in securing mitochondrial health (40). In the present study, we mainly focused on the canonical UPRmt transcriptional axis. The UPRmt transcriptional response involves activating mitochondrial molecular chaperones and quality-control proteases (20). The UPRmt in *C. elegans* is coordinated by multiple factors, including the transcription factor ATFS-1. Haynes et al. (41) confirmed that regulation of the UPRmt is conserved from worms to mammals and that ATF5, the homolog of ATFS-1, is required for UPRmt activation in mammalian cells. In the present study, the data showed that in both Sol and TA muscles, the expression levels of ATF5 and the chaperone HSP60 were increased after HFD feeding. However, the relative fold change in these two proteins was more profound in the Sol than in the TA. The expression of protease ClpP increased only in the Sol. These results indicate that the HFD-induced UPRmt is mainly activated in slow-twitch muscle. Consistent with our results, a recent study demonstrated that the UPRmt could be activated in mouse skeletal muscle by short-term HFD feeding (27). Similarly, in mouse epididymal white adipose tissue (eWAT), the UPRmt was activated after unsaturated fish oil diet (UFD) feeding (42). The reasons for the HFD-induced UPRmt have not yet been confirmed, while some potential mechanisms may be involved in this process. First, it has been shown that a long-term HFD can lead to proteostatic perturbation in skeletal muscle. A very recent study by Fletcher et al. (43) showed that immunoproteasome and total proteasome function are significantly reduced in obese muscle. In addition, it is possible that a HFD could induce the elevation of ROS, which may ultimately activate the UPRmt (20, 44). Additionally, accumulated fatty acids may lead to mitochondrial uncoupling and create mitochondrial stress to induce the UPRmt. In support of this view, a recent study showed that overexpression of uncoupling proteins in neurons of *C. elegans* can induce the UPRmt (45). A HFD is known to induce the expression of uncoupling proteins in skeletal muscle, and the upregulation is more pronounced in slow-twitch muscle fibers (46). This may support the muscle type-dependent activation of the UPRmt by a HFD in the present study.

Normal mitochondrial function is thought to be crucial for ensuring glucose metabolism (16). As one of the mitochondrial quality-control system processes, the UPRmt is considered to be able to maintain mitochondrial homeostasis and restore mitochondrial function (47). In the current study, we found that both in the control and the HFD groups, the malate/glutamate-dependent mitochondrial respiration of fast-twitch muscle is higher than that of slow-twitch muscle. This result is consistent with previous studies (7, 48), the results of which showed that TA has significantly higher O_2_ consumption than Sol and first dorsal interosseus muscle. Notably, preserved mitochondrial respiration was observed only in Sol muscle under HFD conditions. In the TA muscle, mitochondrial respiration significantly declined after HFD intervention. HFD feeding has been reported to impair mitochondrial respiratory function in multiple tissues, including skeletal muscle, and to further induce insulin resistance (49, 50). However, this pathological process has been proven to be muscle fiber type dependent. Consistent with our study, Pinho et al. (51) suggested that the alterations in mitochondrial respiration after HFD feeding occur in a fiber type-dependent manner. In contrast to the soleus muscle, palmitate oxidation in the epitrochlearis (mixed muscle type but mainly fast glycolytic) muscle is significantly lower and increased less than that in the Sol muscle after 8 weeks of HFD feeding (51). Mitochondrial respiratory function relies on OXPHOS status, and evidence suggests that the UPRmt is capable of restoring mitochondrial OXPHOS. Nargund et al. (52) demonstrated that the UPRmt induced by ATFS-1 promotes OXPHOS recovery during mitochondrial stress. They found that ATFS-1 associates with both nDNA and mtDNA under mitochondrial stress and ultimately leads to respiratory recovery by orchestrating OXPHOS component assembly and increasing proteostatic capacity (52). Therefore, compared with that in fast-twitch muscle, the higher activation of the UPRmt in slow-twitch muscle helps to maintain mitochondrial respiration, thus protecting glucose metabolism.

In addition to restoring mitochondrial function, the UPRmt also regulates metabolism by elevating mitochondrial-derived peptide (MDP) production (53). As an identified MDP, MOTS-c is a stress-induced mitochondrial-derived 16-amino-acid peptide encoded by the 12S rRNA sORF in mtDNA. In our study, the expression of MOTS-c in the Sol of HFD rats was significantly higher than that of control rats. In the TA muscle, no significant changes in MOTS-c were observed in the HFD and control groups. The UPRmt is positively correlated with MOTS-c, which is considered a retrograde signal for mitochondria to communicate with the nucleus in response to mitochondrial stress (54). In the present study, we also found that the expression of MOTS-c showed a muscle type-specific pattern in line with UPRmt activation. The metabolic regulatory role of MOTS-c was first identified in the process of gene screening for metabolic regulators of human cells, and that study showed that MOTS-c directly targets skeletal muscle and regulates insulin sensitivity in mice (55). Recently, Reynolds et al. (56) indicated that exercise induced the endogenous MOTS-c level in skeletal muscle, which contributes to elevated lipid utilization capacity. Growing evidence suggests that MOTS-c plays a role in coordinating cellular glucose, mitochondrial, and fatty acid metabolism (55, 57). According to recent research, MOTS-c exhibits a fiber type-specific expression pattern because it is positively associated with slow oxidative fibers. The authors hold the position based on their data that the production of MOTS-c in muscle cells is increased with aging, and it is tempting to further speculate that this phenomenon is tied to the greater expression of MOTS-c in slow-type fibers and the age-related fast-to-slow fiber type transition (58). Taken together, the findings show that compared with that in fast-twitch muscle, the higher level of MOTS-c in slow-twitch muscle, which is positively correlated with the UPRmt, may help to maintain glucose metabolism.

Emerging evidence shows that epigenetic mechanisms are key factors to be considered in the coordinated regulation of skeletal muscle fiber types and metabolic patterns. Based on the results mentioned before, we found that the activation of the UPRmt was different in slow and fast-twitch muscles under the same HFD stress. This disparity may be attributed to the difference in epigenetic status in slow- and fast-twitch muscles. According to fundamental studies, epigenetic modulation plays a profound role in regulating the activation of the UPRmt (32, 59). Therefore, to test whether the different statuses of epigenetic modulation of slow- and fast-twitch muscles contribute to the fiber type-specific activation of the UPRmt, we studied the epigenetic modulations of *ATF5*. Chromatin remodeling has been shown to play a central role in UPRmt activation. Tian Y et al. (59) demonstrated that activation of the UPRmt requires the dimethylation of lysine 3 of histone 3 (H3K9) in the presence of *met-2* and *lin-65*, which leads to a compacted and overall silenced chromatin state, while simultaneously, some chromatin portions remain loose, favoring the binding of UPRmt regulators such as DVE-1. On the other hand, UPRmt activation also requires the conserved demethylases JMJD-3.1 and JMJD-1.2 (32), which reduce chromatin compaction by removing methylation from H3K9 and H3K27, respectively (60, 61). Generally, H3K4me3 is regarded as a transcriptionally positive histone modulation, while H3K27me3 is a transcriptionally inhibitory histone modulation (62). Our data indicated that the enrichment rates of H3K4me3 and H3K27me3 on the *ATF5* promoter in Sol and TA muscles were significantly opposite after HFD feeding. Sol had higher H3K4me3 modulation of the *ATF5* promoter region than TA, while TA had higher H3K27me3 modulation of the *ATF5* promoter after HFD intervention. This is quite similar to the preferentially activated UPRmt in Sol. Emerging evidence indicates that the distribution of active histone modifications, such as H3K4me3, largely differs between fast- and slow-twitch muscles. A recent study described epigenetic profiling between fast/glycolytic and slow/oxidative muscles. That study revealed that the epigenome in Sol muscle was quite different from that in extensor digitorum longus (EDL) muscle, and they accounted for the different myocellular characteristics. The study identified transcription factors with motifs enriched in H3K4me3 peaks, such as MEF2C, PPARA, SOX6 and NFATC2, as they are known to influence differentiation and lipid metabolism in slow/oxidative muscle (63). Other studies have demonstrated that slow-twitch muscles have a unique epigenetic system that regulates gene expression, which might be closely associated with contractile and metabolic properties (64, 65). In summary, this differentiated epigenetic modulation of ATF5 may underlie the mechanism involved in muscle fiber-type-specific UPRmt activation.

## 5 Conclusions

**Figure 7:**
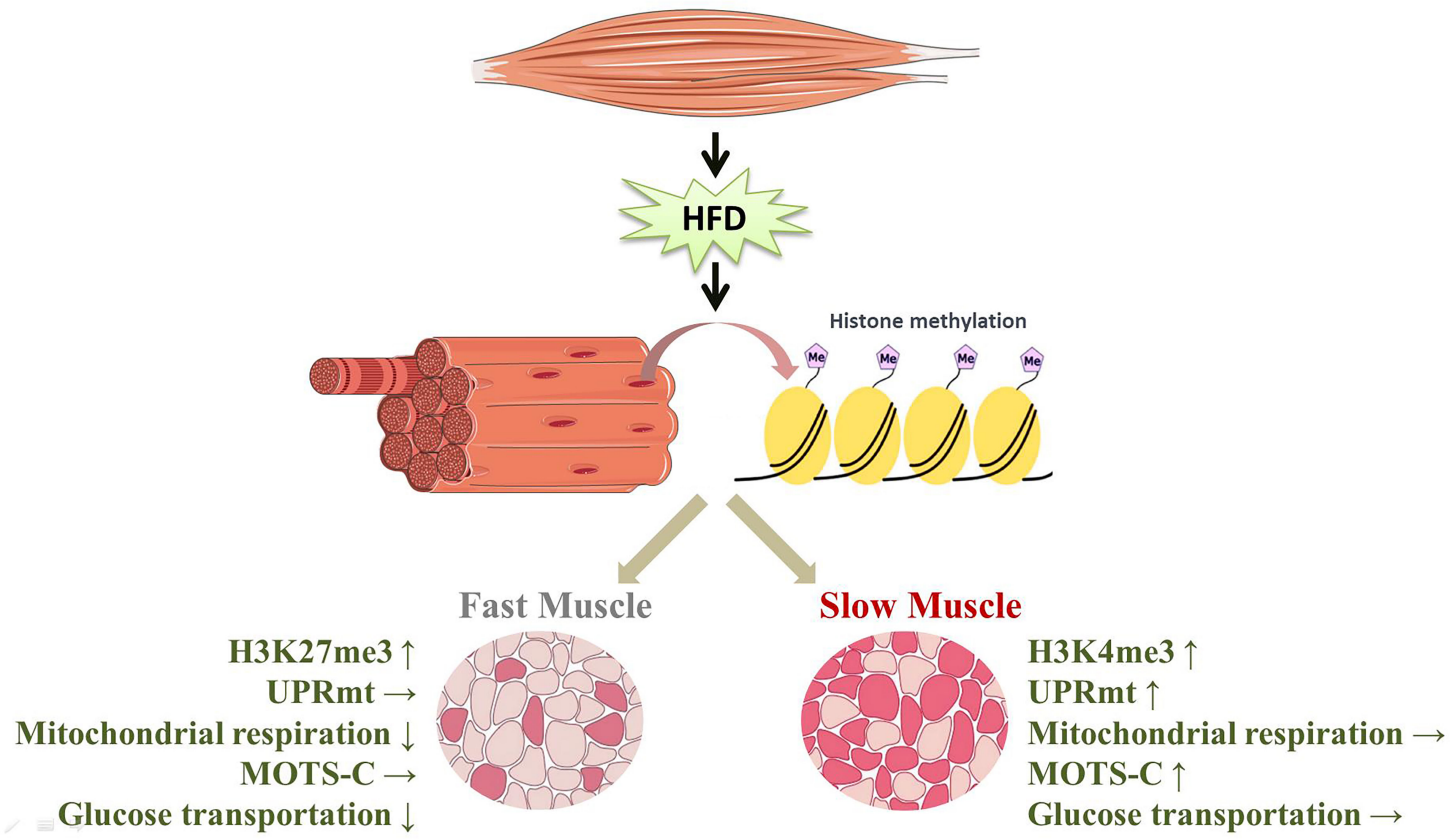
HFD induced the specific activation of the UPRmt in slow- and fast-twitch muscles. (↑, upregulation; ↓, downregulation; →, no change).

In this study, the disparities in the expression of proteins involved in glucose transport show that the glucose transport of slow-twitch muscle remains almost unaltered after HFD intervention, but glucose transport of fast-twitch muscle was significantly impaired. This phenomenon may result from preferential activation of the UPRmt in slow-twitch muscle compared to fast-twitch muscle. On the one hand, due to the mitochondrial repair performed by the UPRmt, mitochondrial respiratory function in slow-twitch muscle is preserved. On the other hand, the higher level of MOTS-c, a UPRmt-related mitokine, may contribute to the maintenance of glucose metabolism in slow-twitch muscle. Notably, this muscle-type-dependent UPRmt activation is probably caused by different histone modifications of the UPRmt regulator (**Figure 7**). However, future work applying genetic or pharmacological approaches should further uncover the relationship between the UPRmt and insulin resistance.

## Data availability statement

The raw data supporting the conclusions of this article will be made available by the authors, without undue reservation.

## Ethics statement

The animal study was reviewed and approved by Ethics Committee of Tianjin University of Sport.

## Author contributions

Conceptualization: CL, HB and YZ. Methodology: CL, NL and ZZ. Investigation: CL and NL. Writing-original draft preparation: CL. Literature research: CL, JL, ZW and YS. Writing-review and editing: CL, HB and YZ. Supervision, review and editing: HB and YZ. All authors contributed to the article and approved the submitted version.

## References

[1] Rolfe DF Brown GC. Cellular energy utilization and molecular origin of standard metabolic rate in mammals. Physiol Rev (1997) 77(3):731–58. doi: 10.1152/physrev.1997.77.3.731 9234964

[2] Gallagher D Belmonte D Deurenberg P Wang Z Krasnow N Pi-Sunyer FX. Organ-tissue mass measurement allows modeling of REE and metabolically active tissue mass. Am J Physiol (1998) 275(2):E249–58. doi: 10.1152/ajpendo.1998.275.2.E249 9688626

[3] Baskin KK Winders BR Olson EN. Muscle as a "mediator" of systemic metabolism. Cell Metab (2015) 21(2):237–48. doi: 10.1016/j.cmet.2014.12.021 PMC4398026 25651178

[4] DeFronzo RA Tripathy D. Skeletal muscle insulin resistance is the primary defect in type 2 diabetes. Diabetes Care (2009) 32 Suppl 2:S157–63. doi: 10.2337/dc09-S302 PMC2811436 19875544

[5] Schiaffino S Reggiani C. Fiber types in mammalian skeletal muscles. Physiol Rev (2011) 91(4):1447–531. doi: 10.1152/physrev.00031.2010 22013216

[6] Yan Z Okutsu M Akhtar YN Lira VA. Regulation of exercise-induced fiber type transformation, mitochondrial biogenesis, and angiogenesis in skeletal muscle. J Appl Physiol (1985). (2011) 110(1):264–74. doi: 10.1152/japplphysiol.00993.2010 PMC3253006 21030673

[7] Crupi AN Nunnelee JS Taylor DJ Thomas A Vit JP Riera CE. Oxidative muscles have better mitochondrial homeostasis than glycolytic muscles throughout life and maintain mitochondrial function during aging. Aging (Albany NY). (2018) 10(11):3327–52. doi: 10.18632/aging.101643 PMC6286850 30449736

[8] Albers PH Pedersen AJ Birk JB Kristensen DE Vind BF Baba O. Human muscle fiber type-specific insulin signaling: impact of obesity and type 2 diabetes. Diabetes. (2015) 64(2):485–97. doi: 10.2337/db14-0590 25187364

[9] Anderson EJ Neufer PD. Type II skeletal myofibers possess unique properties that potentiate mitochondrial H(2)O(2) generation. Am J Physiol Cell Physiol (2006) 290(3):C844–51. doi: 10.1152/ajpcell.00402.2005 16251473

[10] Criswell D Powers S Dodd S Lawler J Edwards W Renshler K. High intensity training-induced changes in skeletal muscle antioxidant enzyme activity. Med Sci Sports Exerc. (1993) 25(10):1135–40. doi: 10.1249/00005768-199310000-00009 8231758

[11] Talbot J Maves L. Skeletal muscle fiber type: using insights from muscle developmental biology to dissect targets for susceptibility and resistance to muscle disease. Wiley Interdiscip Rev Dev Biol (2016) 5(4):518–34. doi: 10.1002/wdev.230 PMC5180455 27199166

[12] Stuart CA McCurry MP Marino A South MA Howell ME Layne AS. Slow-twitch fiber proportion in skeletal muscle correlates with insulin responsiveness. J Clin Endocrinol Metab (2013) 98(5):2027–36. doi: 10.1210/jc.2012-3876 PMC3644602 23515448

[13] Sligar J DeBruin DA Saner NJ Philp AM Philp A. The importance of mitochondrial quality control for maintaining skeletal muscle function across health span. Am J Physiol Cell Physiol (2022) 322(3):C461–C7. doi: 10.1152/ajpcell.00388.2021 35108118

[14] Munoz-Carvajal F Sanhueza M. The mitochondrial unfolded protein response: A hinge between healthy and pathological aging. Front Aging Neurosci (2020) 12:581849. doi: 10.3389/fnagi.2020.581849 33061907 PMC7518384

[15] Anderson EJ Lustig ME Boyle KE Woodlief TL Kane DA Lin CT. Mitochondrial H2O2 emission and cellular redox state link excess fat intake to insulin resistance in both rodents and humans. J Clin Invest. (2009) 119(3):573–81. doi: 10.1172/JCI37048 PMC2648700 19188683

[16] Jheng HF Tsai PJ Guo SM Kuo LH Chang CS Su IJ. Mitochondrial fission contributes to mitochondrial dysfunction and insulin resistance in skeletal muscle. Mol Cell Biol (2012) 32(2):309–19. doi: 10.1128/MCB.05603-11 PMC3255771 22083962

[17] Mogensen M Sahlin K Fernstrom M Glintborg D Vind BF Beck-Nielsen H. Mitochondrial respiration is decreased in skeletal muscle of patients with type 2 diabetes. Diabetes. (2007) 56(6):1592–9. doi: 10.2337/db06-0981 17351150

[18] Teodoro BG Baraldi FG Sampaio IH Bomfim LH Queiroz AL Passos MA. Melatonin prevents mitochondrial dysfunction and insulin resistance in rat skeletal muscle. J Pineal Res (2014) 57(2):155–67. doi: 10.1111/jpi.12157 24981026

[19] Martinus RD Garth GP Webster TL Cartwright P Naylor DJ Hoj PB. Selective induction of mitochondrial chaperones in response to loss of the mitochondrial genome. Eur J Biochem (1996) 240(1):98–103. doi: 10.1111/j.1432-1033.1996.0098h.x 8797841

[20] Zhao Q Wang J Levichkin IV Stasinopoulos S Ryan MT Hoogenraad NJ. A mitochondrial specific stress response in mammalian cells. EMBO J (2002) 21(17):4411–9. doi: 10.1093/emboj/cdf445 PMC126185 12198143

[21] Melber A Haynes CM. UPR(mt) regulation and output: a stress response mediated by mitochondrial-nuclear communication. Cell Res (2018) 28(3):281–95. doi: 10.1038/cr.2018.16 PMC5835775 29424373

[22] Naresh NU Haynes CM. Signaling and regulation of the mitochondrial unfolded protein response. Cold Spring Harb Perspect Biol (2019) 11(6):a033944. doi: 10.1101/cshperspect.a033944 30617048 PMC6546047

[23] Pellegrino MW Nargund AM Kirienko NV Gillis R Fiorese CJ Haynes CM. Mitochondrial UPR-regulated innate immunity provides resistance to pathogen infection. Nature. (2014) 516(7531):414–7. doi: 10.1038/nature13818 PMC4270954 25274306

[24] Wang YT Lim Y McCall MN Huang KT Haynes CM Nehrke K. Cardioprotection by the mitochondrial unfolded protein response requires ATF5. Am J Physiol Heart Circ Physiol (2019) 317(2):H472–H8. doi: 10.1152/ajpheart.00244.2019 PMC6732477 31274354

[25] Smyrnias I Gray SP Okonko DO Sawyer G Zoccarato A Catibog N. Cardioprotective effect of the mitochondrial unfolded protein response during chronic pressure overload. J Am Coll Cardiol (2019) 73(14):1795–806. doi: 10.1016/j.jacc.2018.12.087 PMC6456800 30975297

[26] Gariani K Menzies KJ Ryu D Wegner CJ Wang X Ropelle ER. Eliciting the mitochondrial unfolded protein response by nicotinamide adenine dinucleotide repletion reverses fatty liver disease in mice. Hepatology. (2016) 63(4):1190–204. doi: 10.1002/hep.28245 PMC4805450 26404765

[27] Lee H Ha TY Jung CH Nirmala FS Park SY Huh YH. Mitochondrial dysfunction in skeletal muscle contributes to the development of acute insulin resistance in mice. J Cachexia Sarcopenia Muscle. (2021) 12(6):1925–39. doi: 10.1002/jcsm.12794 PMC8718067 34605225

[28] Antunes LC Elkfury JL Jornada MN Foletto KC Bertoluci MC. Validation of HOMA-IR in a model of insulin-resistance induced by a high-fat diet in wistar rats. Arch Endocrinol Metab (2016) 60(2):138–42. doi: 10.1590/2359-3997000000169 27191048

[29] Clayton DA Shadel GS. Purification of mitochondria by sucrose step density gradient centrifugation. Cold Spring Harb Protoc (2014) 2014(10):pdb prot080028. doi: 10.1101/pdb.prot080028 25275106

[30] Torres MJ Kew KA Ryan TE Pennington ER Lin CT Buddo KA. 17beta-estradiol directly lowers mitochondrial membrane microviscosity and improves bioenergetic function in skeletal muscle. Cell Metab (2018) 27(1):167–79.e7. doi: 10.1016/j.cmet.2017.10.003 29103922 PMC5762397

[31] Feng W Yonezawa M Ye J Jenuwein T Grummt I. PHF8 activates transcription of rRNA genes through H3K4me3 binding and H3K9me1/2 demethylation. Nat Struct Mol Biol (2010) 17(4):445–50. doi: 10.1038/nsmb.1778 20208542

[32] Merkwirth C Jovaisaite V Durieux J Matilainen O Jordan SD Quiros PM. Two conserved histone demethylases regulate mitochondrial stress-induced longevity. Cell. (2016) 165(5):1209–23. doi: 10.1016/j.cell.2016.04.012 PMC4889222 27133168

[33] Chalkley SM Hettiarachchi M Chisholm DJ Kraegen EW. Long-term high-fat feeding leads to severe insulin resistance but not diabetes in wistar rats. Am J Physiol Endocrinol Metab (2002) 282(6):E1231–8. doi: 10.1152/ajpendo.00173.2001 12006352

[34] Morris EM Meers GME Ruegsegger GN Wankhade UD Robinson T Koch LG. Intrinsic high aerobic capacity in Male rats protects against diet-induced insulin resistance. Endocrinology. (2019) 160(5):1179–92. doi: 10.1210/en.2019-00118 PMC6482035 31144719

[35] Pataky MW Wang H Yu CS Arias EB Ploutz-Snyder RJ Zheng X. High-fat diet-induced insulin resistance in single skeletal muscle fibers is fiber type selective. Sci Rep (2017) 7(1):13642. doi: 10.1038/s41598-017-12682-z 29057943 PMC5651812

[36] Sharma M Dey CS. AKT ISOFORMS-AS160-GLUT4: The defining axis of insulin resistance. Rev Endocr Metab Disord (2021) 22(4):973–86. doi: 10.1007/s11154-021-09652-2 33928491

[37] Shortreed KE Krause MP Huang JH Dhanani D Moradi J Ceddia RB. Muscle-specific adaptations, impaired oxidative capacity and maintenance of contractile function characterize diet-induced obese mouse skeletal muscle. PloS One (2009) 4(10):e7293. doi: 10.1371/journal.pone.0007293 19806198 PMC2752162

[38] Turner N Kowalski GM Leslie SJ Risis S Yang C Lee-Young RS. Distinct patterns of tissue-specific lipid accumulation during the induction of insulin resistance in mice by high-fat feeding. Diabetologia. (2013) 56(7):1638–48. doi: 10.1007/s00125-013-2913-1 23620060

[39] Pataky MW Van Acker SL Dhingra R Freeburg MM Arias EB Oki K. Fiber type-specific effects of acute exercise on insulin-stimulated AS160 phosphorylation in insulin-resistant rat skeletal muscle. Am J Physiol Endocrinol Metab (2019) 317(6):E984–E98. doi: 10.1152/ajpendo.00304.2019 PMC6957376 31573845

[40] Munch C. The different axes of the mammalian mitochondrial unfolded protein response. BMC Biol (2018) 16(1):81. doi: 10.1186/s12915-018-0548-x 30049264 PMC6060479

[41] Fiorese CJ Schulz AM Lin YF Rosin N Pellegrino MW Haynes CM. The transcription factor ATF5 mediates a mammalian mitochondrial UPR. Curr Biol (2016) 26(15):2037–43. doi: 10.1016/j.cub.2016.06.002 PMC4980197 27426517

[42] Bhaskaran S Unnikrishnan A Ranjit R Qaisar R Pharaoh G Matyi S. A fish oil diet induces mitochondrial uncoupling and mitochondrial unfolded protein response in epididymal white adipose tissue of mice. Free Radic Biol Med (2017) 108:704–14. doi: 10.1016/j.freeradbiomed.2017.04.028 28455142

[43] Fletcher E Wiggs M Greathouse KL Morgan G Gordon PM. Impaired proteostasis in obese skeletal muscle relates to altered immunoproteasome activity. Appl Physiol Nutr Metab (2022) 47(5):555–64. doi: 10.1139/apnm-2021-0764 35148206

[44] Runkel ED Liu S Baumeister R Schulze E. Surveillance-activated defenses block the ROS-induced mitochondrial unfolded protein response. PloS Genet (2013) 9(3):e1003346. doi: 10.1371/journal.pgen.1003346 23516373 PMC3597513

[45] Shao LW Niu R Liu Y. Neuropeptide signals cell non-autonomous mitochondrial unfolded protein response. Cell Res (2016) 26(11):1182–96. doi: 10.1038/cr.2016.118 PMC5099867 27767096

[46] Schrauwen P Hoppeler H Billeter R Bakker AH Pendergast DR. Fiber type dependent upregulation of human skeletal muscle UCP2 and UCP3 mRNA expression by high-fat diet. Int J Obes Relat Metab Disord (2001) 25(4):449–56. doi: 10.1038/sj.ijo.0801566 11319645

[47] Fiorese CJ Haynes CM. Integrating the UPR(mt) into the mitochondrial maintenance network. Crit Rev Biochem Mol Biol (2017) 52(3):304–13. doi: 10.1080/10409238.2017.1291577 PMC5571472 28276702

[48] Conley KE Amara CE Jubrias SA Marcinek DJ. Mitochondrial function, fibre types and ageing: new insights from human muscle in vivo. Exp Physiol (2007) 92(2):333–9. doi: 10.1113/expphysiol.2006.034330 17170059

[49] Liu R Jin P Yu L Wang Y Han L Shi T. Impaired mitochondrial dynamics and bioenergetics in diabetic skeletal muscle. PloS One (2014) 9(3):e92810. doi: 10.1371/journal.pone.0092810 24658162 PMC3962456

[50] Heyne E Schrepper A Doenst T Schenkl C Kreuzer K Schwarzer M. High-fat diet affects skeletal muscle mitochondria comparable to pressure overload-induced heart failure. J Cell Mol Med (2020) 24(12):6741–9. doi: 10.1111/jcmm.15325 PMC7299710 32363733

[51] Pinho RA Sepa-Kishi DM Bikopoulos G Wu MV Uthayakumar A Mohasses A. High-fat diet induces skeletal muscle oxidative stress in a fiber type-dependent manner in rats. Free Radic Biol Med (2017) 110:381–9. doi: 10.1016/j.freeradbiomed.2017.07.005 28690197

[52] Nargund AM Fiorese CJ Pellegrino MW Deng P Haynes CM. Mitochondrial and nuclear accumulation of the transcription factor ATFS-1 promotes OXPHOS recovery during the UPR(mt). Mol Cell (2015) 58(1):123–33. doi: 10.1016/j.molcel.2015.02.008 PMC4385436 25773600

[53] Durieux J Wolff S Dillin A. The cell-non-autonomous nature of electron transport chain-mediated longevity. Cell. (2011) 144(1):79–91. doi: 10.1016/j.cell.2010.12.016 21215371 PMC3062502

[54] Lee C Kim KH Cohen P. MOTS-c: A novel mitochondrial-derived peptide regulating muscle and fat metabolism. Free Radic Biol Med (2016) 100:182–7. doi: 10.1016/j.freeradbiomed.2016.05.015 PMC5116416 27216708

[55] Lee C Zeng J Drew BG Sallam T Martin-Montalvo A Wan J. The mitochondrial-derived peptide MOTS-c promotes metabolic homeostasis and reduces obesity and insulin resistance. Cell Metab (2015) 21(3):443–54. doi: 10.1016/j.cmet.2015.02.009 PMC4350682 25738459

[56] Reynolds JC Lai RW Woodhead JST Joly JH Mitchell CJ Cameron-Smith D. MOTS-c is an exercise-induced mitochondrial-encoded regulator of age-dependent physical decline and muscle homeostasis. Nat Commun (2021) 12(1):470. doi: 10.1038/s41467-020-20790-0 33473109 PMC7817689

[57] Li S Wang M Ma J Pang X Yuan J Pan Y. MOTS-c and exercise restore cardiac function by activating of NRG1-ErbB signaling in diabetic rats. Front Endocrinol (Lausanne). (2022) 13:812032. doi: 10.3389/fendo.2022.812032 35370955 PMC8969227

[58] D'Souza RF Woodhead JST Hedges CP Zeng N Wan J Kumagai H. Increased expression of the mitochondrial derived peptide, MOTS-c, in skeletal muscle of healthy aging men is associated with myofiber composition. Aging (Albany NY). (2020) 12(6):5244–58. doi: 10.18632/aging.102944 PMC7138593 32182209

[59] Tian Y Garcia G Bian Q Steffen KK Joe L Wolff S. Mitochondrial stress induces chromatin reorganization to promote longevity and UPR(mt). Cell. (2016) 165(5):1197–208. doi: 10.1016/j.cell.2016.04.011 PMC4889216 27133166

[60] Sobue S Inoue C Hori F Qiao S Murate T Ichihara M. Molecular hydrogen modulates gene expression *via* histone modification and induces the mitochondrial unfolded protein response. Biochem Biophys Res Commun (2017) 493(1):318–24. doi: 10.1016/j.bbrc.2017.09.024 28890349

[61] Richards BJ Slavin M Oliveira AN Sanfrancesco VC Hood DA. Mitochondrial protein import and UPR(mt) in skeletal muscle remodeling and adaptation. Semin Cell Dev Biol (2022) 143:28–36. doi: 10.1016/j.semcdb.2022.01.002 35063351

[62] Woo H Ha SD Lee SB Buratowski S Kim TSJE Medicine M. Modulation of gene expression dynamics by co-transcriptional histone methylations. Exp Mol Med (2017) 49(4):e326. doi: 10.1038/emm.2017.19 28450734 PMC6130219

[63] Bengtsen M Winje IM Eftestol E Landskron J Sun C Nygard K. Comparing the epigenetic landscape in myonuclei purified with a PCM1 antibody from a fast/glycolytic and a slow/oxidative muscle. PloS Genet (2021) 17(11):e1009907. doi: 10.1371/journal.pgen.1009907 34752468 PMC8604348

[64] Kawano F Nimura K Ishino S Nakai N Nakata K Ohira Y. Differences in histone modifications between slow- and fast-twitch muscle of adult rats and following overload, denervation, or valproic acid administration. J Appl Physiol (1985). (2015) 119(10):1042–52. doi: 10.1152/japplphysiol.00289.2015 26404615

[65] Kawano F. Histone modification: A mechanism for regulating skeletal muscle characteristics and adaptive changes. Appl Sci (2021) 11(9):3905. doi: 10.3390/app11093905

